# Dr. Williams on the Sounds Produced by the Action of the Heart; with Criticisms

**Published:** 1830-01-01

**Authors:** 


### On the Sounds Produced by the Action of the Heart. By David Williams, M.D. Physician to the North Dispensary, Liverpool.*

Every one at all conversant with auscultation is aware that a double sound is heard in the region of the heart;—the first somewhat prolonged, dull, accompanied with a stroke more or less powerful against the ear or the cylinder, and synchronous with the pulse at the wrist; the second following so hard upon the first as almost to seem like its echo, shorter, clearer, attended with little or no impulsion, and not in correspondence with the arterial pulse. After this second sound there is an interval of repose, broken by the recommencement of a similar order of phenomena. M. Laennec attributed the first to the systole of the ventricle, the second to that of the auricle. Mr. Turner in the third volume of the Transactions of the Medico-Chirurgical Society of Edinburgh endeavoured to overthrow this explanation of M. Laennec's, though he perfectly admitted the correctness of his observation respecting the rhythm of the two cardiac sounds. Mr. Turner allows that the first sound corresponds with the systole of the ventricle, but that the second is produced by that of the auricle he conceives to be inconsistent with the nature of the action of the heart, as ascertained by physiologists, from the inspection of that organ in living animals. The order of events according to M. Laennec is, first the systole of the ventricles, then that of the auricles, and after the *latter* the diastole or period of repose; whereas the experiments of the *vivisectors* place the contraction of the auricle first, and the diastole of the heart after the systole of the *ventricle*. Lancisi supposed that the contractions of the auricles and ventricles were synchronous with each other, and Mr. Turner believes that "the succession of the motions is at all events so instantaneous that it is difficult to distinguish them from each other."

In accordance with this opinion Mr. T. concludes that in applying the ear or the hand to the thorax, the contraction of the auricles either is not evident to the senses, or is perceived so continuous with that of the ventricles that the two communicate only one sensation. But how then to account for the second sound which is heard on applying the ear? The gist of Mr. Turner's

---

* Edin. Med. and Surg. Journ. No. CI. Oct. 1829.

explanation is conveyed in the two following propositions:—1st, "Whether it can be accounted for by the impulse occasioned by the falling back on the pericardium of the relaxed heart in its diastole, after it has been elevated or moved from its place in the systole?" 2ndly, "Whether the expansion of the ventricles immediately after their systole may not contribute in addition to the falling back of the heart?" Both these propositions are so purely hypothetical, and we may add so repugnant to all just physiological reasoning, that we shall not expend our time in controverting them; they fall by their own weight.

Dr. Williams though opposed to Mr. Turner's conjectures, is yet so far staggered by his arguments as to believe that the second sound is not what Laennec imagined it to be, the product of the auricular contraction, but dependent on some other cause. Dr. Williams believes that the systole of the auricle immediately precedes that of the ventricle and that the sounds of the two are blended into one, and also that the diastole succeeds the ventricular contraction. But how then, to repeat our former question, to account for the second sound? Dr. Williams enters into the following explanation which we transcribe entire, as it cannot well be abridged.

"As the cause which I imagine to produce the sound in question appears to warrant a somewhat different explanation of the action of the heart to what has hitherto been suggested by any physiologists whose writings I have perused, I shall make a few observations on the internal appearance and structure of the cavities of this viscus, so as to enable me to explain the ideas which I entertain on the subject more clearly. In examining the interior of the cavities of the right side of the heart, we cannot but be struck with the difference observable between the smooth and uniform surface of the sinus venosus and the reticulated appearance of the auricula. When we contemplate this very marked difference with reference to its purpose in the economy, we must conclude the fleshy fasciculi of the auricula to be calculated for much greater latitude of action than the slightly muscular and even parietes of the sinus venosus. In confirmation of this, if we can rely on the testimony of the eye and touch, we find the contraction of the auricula to be sudden and decidedly distinct from that of the sinus venosus, and also to be much more obvious. To the eye every muscular fibre of the auricula when it contracts is apparently thrown into the greatest degree of action, and appears as if it were contracted to the utmost; to the fingers it also feels as if the opposite sides were brought completely into contact. On the contrary, the action of the sinus venosus seems to the eye to be extremely limited, to be apparently a continual movement; and the touch detects no sudden and active contractions like those of the auricula, nor any thing like an approach to contact of its opposite sides. From this, then, it appears that the auricle (I mean the cavity as including the sinus venosus and auricula,) when the auricula contracts does not empty itself completely of the blood which it contains; and further, that only the auricula can be said to contract actively. A somewhat distant resemblance is observable between the internal surface of the ventricle and that of the auricle. In the ventricle we have a smooth and also a reticulated surface. The smooth and equal channel, which leads towards the arterial orifice, may be compared to the uniform surface of the sinus venosus, and again, the columnæ carneæ to the musculi pectinati. As the auricle most probably propels only a part of the blood which it contains into the ventricle at each contraction of the auricula, it is not unreasonable to suppose that the ventricle likewise propels only a portion of its blood into the artery at each systole. Since then a quantity of blood is probably left after each systole, we may I think infer from the uniformity of the surface of the channel leading towards the arterial orifice, that this channel is the principal lodgement of the unexpelled blood.

"The valves of the auricular and arterial orifices of the heart, although they exercise similar offices, yet in their mechanism differ materially; and if we may judge from the properties of their respective structures, we may, I think, infer the immediate power by which they exercise their offices to be partially of a very different nature. The semilunar valves are simple membranous folds, and consequently exercise their offices independently of any contractile property inherent in themselves;—they are closed and opened by the direct impulse of the alternate currents of the blood. Their action may therefore be considered to be as purely a mechanical one as any in the body. The auricu'o-ventricular valves are likewise membranous folds, but they have inserted along their fringed edges the tendons of the musculi papillares. As the latter form a part of the valvular apparatus, and have inherent in themselves the principle of motive power, the valves of the auricular orifices must necessarily exercise their office in a different manner from those of the arterial orifices. The contractile property inherent in the structure of the auricular valves has I think engaged but little of the attention of physiologists, since their action seems to be regarded as equally mechanical as that of the semilunar valves. The musculi papillares appear to be considered as processes having little or no action,—as in fact nothing more than a stay to prevent the valve when shut from being pushed into the auricle, for the valve is assumed to be opened by the impulse of the blood in its passage from the auricle into the ventricle. Mr. John Bell, in explaining the manner in which he supposes the function of the auricular valves to be exercised, states, that 'the valves fall down easily when the blood goes down through them, and they rise readily and quickly whenever the blood gets behind them.' And he adds, 'the auricle by its action lays down the tricuspid or auricular valve and fills the ventricle.'* In these extracts it is evidently implied that the auricular valves are both opened and closed solely by the impulse of the blood. That the auricular valve is closed solely by the impulse of the blood cannot be doubted; but, with every deference, I certainly must differ in opinion with respect to the power which opens it. As we cannot observe the manner in which the auriculo-ventricular valves perform the valvular office, I must beg particular attention to the state we find them in; to the appearance of the musculi papillares; and to their probable condition at the moment when the systole of the heart is terminating. In examining the inner surface of the ventricular cavity we see the valve descending contiguous to the sides of the ventricle, and we find the chordæ tendineæ to be dense to the touch. The musculi papillares we observe to be distinct muscular columns, of different sizes, and extremely compact in their structure. From the tense state of the tendinous chords, and the total want of any other mechanism to prevent the valves from being pushed into the auricle, it is pretty evident that the valve cannot close the orifice and resist the force of the systole of the ventricle without the musculi papillares being extended. If then the musculi papillares be extended by the impulse of the blood during the systole of the ventricle, it is obvious that their extreme state of extension must be at the instant the systole is terminating.

"That the musculi papillares are subservient to the action of the auricular valves is not questioned. But those authors whose writings I have perused seem to think, as I have already stated, that they act only as a stay to prevent the valve from being pushed into the auricle; for they consider the valve itself to be opened by the impulse of the blood in its passage from the auricle into the ventricle. Now, it is evident, since the resistance must be equal to the impulse of the force applied, that the musculi papillares cannot act as a stay to the valve against the very powerful impulse which must be communicated at each systole, without being extended, and if extended, it follows that they must contract. Now, if we may judge the

* "Bell's Anatomy, Article Heart."

contraction of the musculi papillares to be any way in proportion to their size and compactness, to the strength of their tendons, and to the power of their antagonist, we shall then undoubtedly have a cause fully equal to produce the sound in question.

"My reason for supposing the closing of the semilunar valves to have little to do in causing the sound I am endeavouring to account for is, 1*st*, because they have very limited scope for motion; 2*dly*, because the power which causes the reflux of blood is simple reaction; 3*dly*, because in closing their motion must be somewhat moderated by the pressure of the unexpelled blood yet remaining in the ventricle. For these reasons I am inclined to think that the sound which results from the closing of the semilunar valves in health is so inconsiderable as to be entirely drowned by that arising from the opening of the auricular valves. It is then by the contraction of the musculi papillares that I am disposed to think the auriculo-ventricular valves to be mainly *opened;* and the stroke of the valves against the sides of the ventricles, in their rapid retraction, I conceive to be the cause of the second of the two successive successions or sounds heard in exploring the action of the heart.

"I shall now offer a few remarks on the action of the heart. From what I have already stated, it appears to me, 1*st*, That the auricula is the only portion of the auricle which contracts actively; 2*dly*, That the contraction of the sinus venosus is very limited, and of a passive nature; 3*dly*, That the auricle and ventricle expel only a part of the blood which they respectively contain at each contraction; 4*thly*, That the principal lodgement of the unexpelled blood in the auricle, when the auricula contracts, is the sinus venosus, and in the ventricle, at the termination of the systole of the heart, the channel which leads to the arterial orifice. Let us suppose the right ventricle to be contracting, the blood it contains being strongly pressed raises the tricuspid valve, and its divisions shut the auriculo-ventricular orifice; also it opens the semilunar valves, and a wave of blood passes into the artery. At the instant the contraction of the heart is ceasing, the semilunar valves are in the fossæ valsalvæ, the tricuspid is closed, and the musculi papillares, in resisting the pressure of the blood from pressing the valve into the auricle, are in a state of extension. At the same moment that the contraction of the heart is ceasing, the musculi papillares contract, and by their contraction open the tricuspid valve. Likewise, as soon as the re-action of the artery causes a reflux of blood towards the ventricle, the semilunar valves are raised and closed by the pressure of the blood. If we may judge from analogy, there can be no doubt but that the retraction of the auricular valve is extremely rapid, consequently it must draw along with it a portion of blood from the auricle into the ventricle; and further, the blood lodged between its surface and the sides of the ventricle must be forcibly impelled towards the interior of the cavity. What quantity of blood may be drawn into the ventricle at each contraction of the musculi papillares is a question of no easy solution. However, the resistance which otherwise would be opposed to the passage of the blood from the auricle into the ventricle must be materially lessened by the effect produced by the retraction of the valve. It may be asked, how far the influence of the contraction of the musculi papillares affects the circulation? The effect produced by the retraction of the valves is certainly a source of actual power, that is, motion is generated. Though we cannot ascertain the quantity of blood drawn into the ventricle at each contraction, yet I think we may infer the influence of its operation to be strictly local; for, from the pressure with which the blood is pressed towards the auricle, it appears to me to be very evident that the blood-vessels are in need of no aid to assist them in returning the blood into the sinus venosus; therefore, under this circumstance, any aid would be superfluous. From this view of the subject, I am inclined to consider the influence of the motion generated by the contraction of the musculi papillares to be, as a power of derivation, of no greater service in aiding the vessels to return the blood into the sinus venosus, than the influence of the contraction of the auricula; both contributing simply to the passage of the blood from the auricle into the ventricle. Accordingly, I consider the effect produced by the retraction of the valve of the auricular orifice to be immediately subordinate to the function of the auricle. The musculi papillares, first of all, by retracting the valve, draw along with it a portion of blood into the ventricle: then another portion is impelled into it by the reaction of the sinus venosus, together with the impulse of the blood from the venæ cavæ; and lastly, to complete the requisite extension of the ventricle, the auricula contracts, and instantly, for no perceptible time intervenes, the systole of the heart follows; when again the musculi papillares contract, and so the succession goes on."

The foregoing theory is very laboured, very ingenious, and very incorrect. It is a pity that when Dr. Williams was reviewing the anatomical construction of the heart with such scrutinizing eye, he did not look at those auriculo-ventricular valves whose function he mistakes. Dr. Williams argues boldly that the musculi papillares or columnæ carneæ *open* the valves; it is rather unfortunate for him that they *close* it. Take, for instance, the mitral valve:—its two alæ are not supplied by separate packets of musculi papillares, which if so arranged might by possibility pull those two alæ asunder, and open the valve; no! one batch of the columnæ carneæ sends off chordæ tendineæ which diverge and supply *the opposite edges of either ala,* thus obviously and beautifully operating to approximate them and thereby close the valve.* This is a plain anatomical fact which Dr. Williams ought to have known, and which if known would have saved him the trouble of writing more than eight pages of close letter-press to prove his error. The mistake is so palpable that had it not been made by a writer of Dr. Williams's celebrity we should never have thought it worth correction. As however his authority might lead some astray we have thought it our duty to strangle the misconception in embryo, lest it should grow into a mischievous maturity. It is needless to observe that the foundation being thus withdrawn from beneath its feet, the whole series of speculations falls at once into the abyss of nothingness. We have only in conclusion to express our belief that the second sound heard on exploring the heart is what Laennec unhesitatingly asserted it to be, that of the auricular systole, and that this belief has not yet been shaken by the arguments of Mr. Turner or the theories of Dr. Williams.

* Even if this direct arrangement were absent, there are many unanswerable reasons for the musculi papillares closing the valve during the ventricular systole. As it is, we should only be wasting our reader's time to advance them, when the anatomy tells its own tale so clearly.—*Rev.*

